# Supplementary material for: Glycated Albumin, a Novel Biomarker for Short-Term Functional Outcomes in Acute Ischemic Stroke
Source: Brain Sci. 2021 Mar 6;11(3):337. doi: 10.3390/brainsci11030337 (PMC8000654; doi:10.3390/brainsci11030337)
Supplement: Supplementary file 1 [file brainsci-11-00337-s001.pdf]

Supplementary Material

Table S1. Correlations between body mass index and glycemic control indicators.

	**BMI, Q1**	**BMI, Q2**	**BMI, Q3**	**BMI, Q4**	***p*-Value**
No. (%)	331 (24.6)	328 (24.4)	339 (25.2)	333 (24.7)	
Age, years	72 ± 14 [a]	71 ± 13 [a]	68 ± 12 [b]	67 ± 13 [b]	**<0.001 [1)]**
Diabetes	85 (29.3)	94 (33.7)	109 (36.9)	118 (41.4)	**0.002 ***
Laboratory					
FBS, mg/dL	130.3 ± 53.6 [a]	139.4 ± 66.7 [a]	132.4 ± 50.5 [a]	140.6 ± 58.1 [a]	0.109 [1)]
Initial glucose, mg/dL	139.1 ± 56.0 [a]	145.5 ± 65.3 [a]	143.2 ± 56.3 [a]	150.5 ± 62.0 [a]	0.185 [1)]
HbA1c, g/dL	5.99 ± 1.19 [a]	6.25 ± 1.39 [a,b]	6.25 ± 1.34 [a,b]	6.35 ± 1.36 [b]	**0.004 [1)]**
HbA1c ≥6.5%	61 (21.4)	72 (25.9)	82 (27.9)	100 (35.2)	**<0.001 ***
Glycoalbumin, Quartiles					**0.003 ***
Glycoalbumin, 1Q	47 (16.3)	56 (20.1)	90 (30.5)	78 (27.4)	
Glycoalbumin, 2Q	72 (25.0)	79 (28.3)	64 (21.7)	76 (26.7)	
Glycoalbumin, 3Q	93 (32.3)	74 (26.5)	70 (23.7)	57 (20.0)	
Glycoalbumin, 4Q	76 (26.4)	70 (25.1)	71 (24.1)	74 (26.0)	
GA/HbA1c	2.77 ± 0.51 [a]	2.69 ± 0.48 [b]	2.58 ± 0.39 [b]	2.57 ± 0.41 [b]	**<0.001[1)]**

Abbreviation: BMI, Body mass index; FBS, Fasting blood sugar; IQR, Interquatile ratio. No. (%) or mean ± SD. *p*-Values were calculated by χ^2 test for trend in proportion. * Linear by linear association for trend. [1)] Statistical significances were tested by Oneway analysis of variances among groups. [a,b)] The same letters indicated non-significant difference between groups based on Scheffe multiple comparison test.
